# Niemann–Pick disease type C: introduction and main clinical features

**DOI:** 10.1007/s00415-014-7382-z

**Published:** 2014-08-22

**Authors:** A. Burlina

**Affiliations:** Neurology Unit, St. Bassiano Hospital, Via dei Lotti 40, 36061 Bassano del Grappa, Italy

Niemann–Pick type C (NP-C) is a rare neurodegenerative lysosomal storage disorder with autosomal recessive inheritance that can present during infancy, childhood or adulthood [4, 5, 9]. The highly heterogeneous clinical presentation of NP-C makes diagnosis particularly difficult, and as a consequence, the disease may go undetected or be misdiagnosed. The true prevalence of NP-C is therefore difficult to measure, but it has been estimated at 1 case in every 120,000 live births [9].

NP-C is caused by mutations in either the *NPC1* gene (in approximately 95 % of cases) or the *NPC2* gene (in approximately 4 % of cases). Both types of mutations lead to an intracellular lipid trafficking defect and autophagic dysfunction, with subsequent accumulation of cholesterol, glycosphingolipids, phospholipids and sphingomyelin to varying degrees in the spleen, liver and central nervous system [1, 7, 8]. An extensive review of both the visceral and neurological clinical manifestations of NP-C was published recently by Mengel et al. [3]. Table 1 summarises the main clinical signs and symptoms.

**Table 1:** Clinical findings and symptoms of NP-C, not according to frequency (modified from Mengel et al. [3])

Type	Findings/symptoms
Systemic	Isolated unexplained splenomegaly
	Hepatomegaly/splenomegaly
	Prolonged neonatal cholestatic jaundice
	Hydrops fetalis or foetal ascites
	Aspiration pneumonia, alveolar lipidosis, interstitial lung involvement
	Low HDL cholesterol
	Low serum ceruloplasmin
	Mild thrombocytopenia
Neurological	Vertical supranuclear gaze palsy
	Ataxia
	Dysarthria
	Clumsiness
	Cerebellar syndrome, including all above signs
	Dysphagia
	Dystonia
	Hypotonia
	Gelastic cataplexy
	Hearing loss
	Seizures
	Sleep disturbances (mainly insomnia)
	Delayed developmental milestones
Psychiatric	Cognitive decline
	Organic psychosis
	Disruptive/aggressive behaviour
	Progressive development of treatment-resistant psychiatric symptoms

Analysis of the literature indicates that NP-C diagnoses are often delayed after initial symptom onset, usually by 5–10 years [4, 5]. Despite vast advances in the understanding of the pathogenesis and natural history of the disease over the past 2 decades, the broad spectrum of neurological manifestations, many of which are non-specific, and the variable age of onset present significant hurdles to diagnosis. Table 2 lists the most frequent diseases reported in the literature that, at onset, have been considered as possible diagnoses instead of NP-C.

**Table 2:** Differential diagnoses

Neurological diseases often diagnosed instead of NP-C, as reported in the literature (not according to frequency):
Alzheimer’s disease and frontotemporal dementia
Creutzfeldt–Jakob disease
Multiple sclerosis
Parkinson disease/Parkinsonism
Progressive supranuclear palsy
Psychotic syndromes
Spinocerebellar ataxias
Wernicke encephalopathy
Wilson disease

A valuable tool for the detection and diagnosis of NP-C is the NP-C Suspicion Index, which is based on the prediction value of individual symptoms as well as of their combinations summarised in clusters of visceral, neurological and psychiatric symptoms [10]. This algorithm can help clinicians to identify patients with a high likelihood of NP-C, enabling further diagnostic work up to achieve a diagnosis of NP-C [11].

**Fig. 1:**
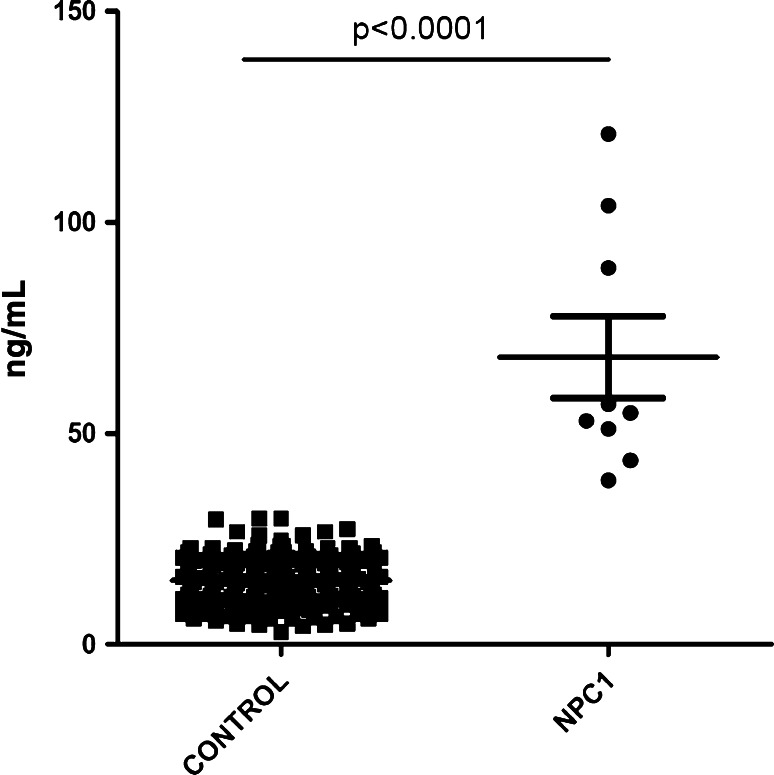
Concentration of endogenous cholestane-3β,5α,6β-triol in plasma of NPC1 patients (*n* = 9; age range 1–55 years) and controls (*n* = 152; age range 18–65 years). Cholestane-3β,5α,6β-triol values for controls and patients are 3–30 and 39–121 ng/mL, respectively. Data provided courtesy of Dr. Giulia Polo

After recording a detailed medical history, and comprehensive clinical and neurological examinations, laboratory diagnostic processes necessary to confirm a diagnosis of NP-C include histological staining with filipin and *NPC1* and *NPC2* gene sequencing. Until recently, assays of suggested plasma markers for NP-C (chitotriosidase, CCL18/PARQ) have not provided consistent findings [4]. However, recent data showing that plasma levels of cholesterol oxidation products (oxysterols) such as cholestane-3β,5α,6β-triol are appreciably higher in NP-C patients compared with controls indicate that oxysterols may serve as sensitive and specific markers for NP-C [2, 6] (Fig. 1).

While brain magnetic resonance imaging or spectroscopy has been shown to be useful for defining or monitoring the presence or progression of neurological disease in NP-C, these imaging techniques do not show a specific pattern of abnormalities that can be considered characteristic of the disease. For instance, bilateral cerebellar atrophy is a common finding in many conditions associated with ataxia. Imaging studies are therefore not generally considered useful in differential diagnosis with respect to NP-C [5].

It is crucial to confirm a definite diagnosis of NP-C as early as possible in suspected cases for two reasons: (1) to ensure best practice for the multidisciplinary clinical management of this complex disease; and (2) because a disease-specific therapy has been available for paediatric and adult patients in Europe since 2009. As recommended in the international guidelines for the clinical management of NP-C, miglustat, the only available approved treatment, should be initiated as soon as possible after confirmed diagnosis to slow or prevent the neurological progression of the disease [4, 5].

The aim of this supplement of the *Journal of Neurology* is to help neurologists to consider NP-C as a possible diagnosis among the major neurodegenerative disorders, and to provide sufficient detail to help achieve a diagnosis. As an increasing number of adolescent and adult-onset cases are being identified and diagnosed, often based on psychiatric symptoms in combination with ataxia and vertical supranuclear gaze palsy, we focus mainly on the clinical manifestations of the disease in adults.

A common theme throughout each of the following articles is the broad spectrum of possible differential diagnoses. Due to the heterogeneity and non-specificity of many of the neurological and neuropsychiatric signs and symptoms associated with NP-C, there is considerable overlap with a range of other inherited metabolic disorders that feature neurological degenerative changes affecting the cortex, brainstem and cerebellum, such as spinocerebellar ataxias, progressive supranuclear palsy and hexosaminidase deficiencies (e.g. Tay–Sachs disease).

Michelangelo Mancuso of the University of Pisa, Italy provides a comprehensive review addressing the complexity of hereditary cerebellar ataxias (CAs). A wide range of genetic defects, including NP-C, are associated with CAs that present in the clinic, often in combination with other neurological symptoms. Dr. Mancuso provides a guide to the diagnosis of underlying disease aetiologies associated with CAs.

Michael Strupp and co-authors from the University of Munich, Germany discuss a clinical approach to the differential diagnosis of central ocular disorders. Abnormal saccadic eye movements, particularly vertical supranuclear gaze palsy, are a widely recognised characteristic neurological sign associated with NP-C, and are often one of the first diagnostic clues for the detection of the disease.

Kirsten McKay from the Midlands Regional Genetics Service, and Paul Gissen from the UCL Institute of Child Health in the UK review the state of the art for laboratory diagnosis of NP-C, with a particular focus on the growing role of genetic sequencing. The development of new approaches to screening and diagnosis is covered, including assays of plasma cholesterol oxidation products (oxysterols) and urinalysis of SNAG-based bile acid constituents to measure cholesterol elimination.

Finally, Saba Nia of the Rosenhügel Neurological Center, Vienna, Austria provides an overview of the pathogenesis, clinical and psychiatric symptomatology and available therapies for treatable inborn errors of metabolism in which patients can initially present with psychiatric signs and symptoms. This is a particularly important aspect to consider when differential diagnoses incorporate NP-C, as patients with adolescent or adult-onset NP-C frequently exhibit cognitive or psychiatric signs, or both, at initial presentation.
